# Deletion of Card9 eliminates the detrimental facets of mycobacterial adjuvants

**DOI:** 10.1016/j.heliyon.2024.e38139

**Published:** 2024-09-19

**Authors:** Hideo Mitsuyama, Ei'ichi Iizasa, Akiko Kukita, Shuji Toda, Hiroki Yoshida, Hiromasa Inoue, Hiromitsu Hara

**Affiliations:** a Department of Pulmonary Medicine, Graduate School of Medical and Dental Sciences, Kagoshima University, 8-35-1 Sakuragaoka, Kagoshima-City, Kagoshima, 890-8544, Japan; b Department of Immunology, Graduate School of Medical and Dental Sciences, Kagoshima University, 8-35-1 Sakuragaoka, Kagoshima-City, Kagoshima, 890-8544, Japan; c Department of Psychosomatic Internal Medicine, Graduate School of Medical and Dental Sciences, Kagoshima University, 8-35-1 Sakuragaoka, Kagoshima-City, Kagoshima, 890-8544, Japan; d Research Center of Arthroplasty, Faculty of Medicine, Saga University, 5-1-1 Nabeshima, Saga-City, Saga. 849-8501, Japan; e Division of Pathology, Department of Pathology and Microbiology, Faculty of Medicine, Saga University, 5-1-1 Nabeshima, Saga-City, Saga, 849-8501, Japan; f Department of Pathology, Takagi Hospital, Okawa, Fukuoka, 831-8501, Japan; g Division of Molecular and Cellular Immunoscience, Department of Biomolecular Sciences, Faculty of Medicine, Saga University, 5-1-1 Nabeshima, Saga-City, Saga, 849-8501, Japan

**Keywords:** Card9, Mycobacteria, Adjuvant, Vaccine, Autoimmunity

## Abstract

Although mycobacterial adjuvants are capable of eliciting a strong adaptive humoral and cellular immunity, they also sometimes provoke detrimental outcomes, including autoimmune/inflammatory syndromes. Here, we show that the deletion of caspase recruitment domain family member 9 (Card9), a signaling adaptor of a set of innate immune receptors, can eliminate the detrimental effects of mycobacterial adjuvants. Long-lasting tissue-destructive skin inflammation at the site of complete Freund's adjuvant (CFA) injection, lung granuloma formation induced by intratracheal *Mycobacterium bovis* Bacillus Calmette-Guérin infection, and the incidence and severity of experimental autoimmune encephalomyelitis and collagen-induced arthritis induced by autoantigen immunization with CFA were considerably attenuated in Card9-deficient (*Card9*^*−/−*^) mice compared to control wild-type mice. *Card9*^*−/−*^ mice showed impaired development of Th17, but not Th1, in the early phase after autoimmune induction, due to the impaired development of IL-6-producing Sirpα^high^ dendritic cells, which are essential for priming pathigenic Th17, in the draining lymph nodes. However, Card9 deletion did not affect overall adaptive antibody production or delayed-type hypersensitivity following immunization with CFA, indicating that humoral and type 1 immune responses remained intact. These results suggest that avoiding the activation of Card9 signaling during vaccination with mycobacteria-containing vaccines may mitigate the risk of detrimental type 3 immune responses, while preserving type 1 immune responses that are effective against intracellular pathogens and cancers.

## Highlights

• Card9 deletion attenuates tissue-damaging inflammation by mycobacterial adjuvants.
• Card9 deletion prevents autoimmune development induced by mycobacterial adjuvant.
• Card9 is required for the early development of pathogenic Th17 for autoimmunity.
• Card9 deletion impairs the development of IL-6-producing Sirpα^high^ dendritic cells.
• Card9 deletion does not affect adaptive humoral and type I cellular immunity induced by mycobacterial adjuvant.

## 1 Introduction

Although adjuvants are indispensable for vaccines to prime effective adaptive immunity to target antigens and pathogens, they sometimes induce undesirable side effects, such as autoimmune/inflammatory syndromes [1]. Therefore, there is a need to strike a balance between beneficial and adverse effects simultaneously, which can be a challenge. Alum (hydroxyaluminium) is the most widely accepted adjuvant for human use. Although alum has contributed to the success of most current vaccines, it is predominantly known to induce humoral immunity, in contrast to its poor induction of classical cell-mediated immunity through CTL responses [2]. Adjuvants that provide a stronger immune response are becoming increasingly important for combating infectious pathogens and cancers, with a new generation of vaccine candidates that include more highly purified antigens. Research is ongoing to identify the best substance or combination of substances for adjuvants to induce the correct humoral and cellular immune responses to the target antigen without causing adverse reactions.

Attenuated mycobacteria, such as *Mycobacterium bovis* Bacillus Calmette-Guérin (BCG), or mycobacterium-derived components, such as cord factor (also known as trehalose di-mycolate (TDM)), lipomannan, heat shock proteins (HSPs), and muramyl-dipeptide (MDP), have been studied as adjuvants in antimicrobial and anticancer vaccines due to their ability to elicit strong humoral and protective immunity [[3], [4], [5], [6], [7]]. Intravesical BCG instillation is the mainstay adjunctive therapy for superficial bladder cancer. Although usually well-tolerated, local and systemic infectious complications, including pulmonary complications with miliary tuberculosis and caseating granulomas, can occur after instillation in the absence of detectable BCG [8]. Complete Freund's adjuvant (CFA), containing heat-killed *Mycobacterium tuberculosis* (Mtb), can induce a strong and sustained immune response and has been successfully used to induce protective immunity against a wide range of pathogens and diseases [9]. Despite its potential benefits, the use of CFA in humans is limited because of its adverse effects. CFA can cause severe inflammation and tissue damage at the injection site as well as systemic immune activation and toxicity. It has also been used as an adjuvant to break self-tolerance and develop autoimmune diseases in animals, including in murine models of multiple sclerosis and rheumatoid arthritis, induced via immunization with tissue-specific autoantigens. Therefore, research into new adjuvant formulations as alternatives to CFA is growing, which allow for a reduced toxicity while maintaining comparable adjuvanticity [10].

Given that adjuvants activate innate immunity via pattern recognition receptors (PRRs), the benefits and side effects of adjuvants can be considered as the result of the activation of specific intracellular signals downstream of these receptors [11]. Therefore, identifying the signaling pathways that lead to favorable or deleterious immune responses to adjuvants is necessary for the design of effective vaccines. Caspase recruitment domain family member 9 (Card9) is a signaling adaptor molecule that mediates the activation of myeloid cells through multiple PRRs, such as ITAM-coupled C-type lectin receptors (CLRs), nucleotide-binding domain and leucine-rich repeat–containing receptors (NLRs), and Toll-like receptors (TLRs) [12]. In particular, the critical role of Card9-mediated innate immune activation by CLRs coupled with spleen tyrosine kinase (Syk) in antifungal and anti-Mtb defense has been demonstrated in mouse models and humans [[13], [14], [15]]. Studies on antifungal immunity suggest that Syk-CARD9 signaling is required for the coupling of innate immune activation with the induction of Th17-mediated type 3 immune responses that are effective against fungal pathogens [14,15]. However, the mechanisms underlying this coupling are not yet fully understood.

In the present study, we demonstrated the effect of Card9 deletion on the favorable and deleterious effects of mycobacterial adjuvants. In Card9-deficient (*Card9*^*−/−*^) mice, long-lasting skin inflammation at the site of CFA injection was alleviated and shortened compared with that in wild-type (WT) mice. In addition, lung granuloma formation after BCG infection was not observed in *Card9*^*−/−*^ mice. The incidence and severity of experimental autoimmune encephalomyelitis (EAE) and collagen-induced arthritis (CIA) induced by CFA were markedly alleviated in *Card9*^*−/−*^ mice, with reduced development of Th17, but not Th1, in draining lymph nodes (dLNs) in the early phase of immunization. We found that Card9 was required to induce IL-6-producing Sirpα^high^ dendritic cells in the dLNs, which was essential for the priming of pathogenic Th17 during autoimmune induction. However, Card9 deficiency did not affect the adaptive humoral and type 1 cellular immune responses induced by an immunization with CFA. Therefore, our findings suggest that the inhibition of Card9 signaling during vaccination may eliminate unwanted type 3 immune responses induced by mycobacterial adjuvants, while retaining the ability to induce type I immune responses that are effective against intracellular pathogens.

## 2 Materials and methods

### 2.1 Reagents and medium

CFA was prepared by mixing incomplete Freund's adjuvant (IFA) (BD Difco, 63-6532-70) with 10 mg/ml of heat-killed Mtb strain H37Ra (BD Difco, 63-6352-68). Chicken type II collagen (c-9301), ovalbumin (OVA) (A5503), lipopolysaccharide (LPS) (*Escherichia coli* O111:B4, L3024), phorbol 12-myristate 13-acetate (PMA) (P8139), ionomycin (19657), Percoll (P1644), and bovine serum albumin BSA (fraction V, A5611) were purchased from Sigma-Aldrich. CpG-ODN (tlrl-1585) was obtained from InvivoGen. MOG_35-55_ peptide (MEVGWYRSPFSRVVHLYRNGK) was synthesized by Eurofins. Pertussis toxin (180) was obtained from List Biological Laboratories. Horseradish peroxidase (HRP)-conjugated goat anti-mouse IgG-HRP (H + L) was purchased from Sigma (12–349). Horseradish peroxidase-conjugated goat anti-mouse IgM, IgG1, and IgG2b antibodies were purchased from Southern Biotech (5300-05). Collagenase Type 4 was obtained from Worthington (CLS-4). ELISA kits for mouse IL-6 (43135), mouse IFN-γ (430815), mouse IL-17A (432504), and mouse IL-12p40 (431604) were obtained from BioLegend. Mouse complement C5a ELISA kit (ab193718) was obtained from Abcam. Anti-mouse CD3ε (145−2C11), anti-mouse CD16/CD32 antibody (2.4G2), anti-mouse CD11b FITC antibody (M1/70), anti-mouse Ly6G PE antibody (1A8), anti-mouse CD45.2 APC (104), anti-mouse CD11c PE (N418), anti-CD172a (SIRPα) APC-Cy7 (P84), anti-mouse IFN-γ FITC (XMG1.2), and anti-mouse IL-17A PE (TC11-18H10.1) were obtained from BioLegend. Anti-mouse CD4 APC (RM4-5), anti-mouse CD4 PE (GK1.5), and anti-I-A/I-E (MHC II) APC (M5/11) antibodies were purchased from TONBO. The anti-PE MicroBeads (130-048-801), MS columns (130-042-201), and LS columns (130-042-401) were purchased from Miltenyi Biotec. The Cytofix/Cytoperm GoldiStop kit (555028) was purchased from BD Biosciences. TMB reagent (ML1120T) was purchased from SUMILON. ACK lysis buffer (0.15 M NH_4_Cl, 10 mM KHCO_3_, 0.1 mM EDTA, adjusted to pH 7.2 1 M HCl) was prepared. For the cell cultures, RPMI1640 (Nacarai Tesque, 30264) supplemented with 10 % FBS (Corning, 35-015), 50 μM of 2-mercaptoethanol (Nacalai Tesque, 21438), and Antibiotic-Antimycotic Mixed Solution (Nacalai Tesque, 09366-44) was used. The vaccine strain of *M. bovis* BCG (Tokyo 172 strain) was obtained from the Japan BCG Laboratory.

### 2.2 Mice and animal experiments

The mice used in this study were aged between 6 and 12 weeks. C57BL/6N mice were purchased from Kyudo Co. *Card9*^*−/−*^ mice were generated as described previously [16]. They were maintained at the animal facility at the Institute of Laboratory Animal Science Research Support Center Kagoshima University in a specific pathogen-free conditions room with a 12-h light/dark cycle at 22 ± 1 °C and 50 ± 10 % humidity. All experiments involving mice were approved by the Animal Research Committee of Kagoshima University, and the animals were treated in accordance with the ethical guidelines of Kagoshima University.

### 2.3 Local and systemic inflammation

To evaluate local inflammation at the injection site of CFA, the mice were subcutaneously injected with 20 μl of CFA into the footpads. Fourteen days after the injection, foot tissues were collected, minced into small pieces with a scissor, and incubated with 0.5 mg/ml of collagenase type 4 in PBS at 37 °C for 2 h with occasionally mixing by vortex. After filtration through a 70-μm nylon cell strainer (Falcon, 352350), the cells were washed and hemolyzed using ACK buffer. After washing, the cells were dissolved in 5 ml of 35 % Percoll solution (diluted in 1 × PBS) and centrifuged at 2000 rpm for 20 min at room temperature. The resulting pellets were collected and immune cells were analyzed using flow cytometry. For the flow cytometric analysis of the infiltrated macrophages and neutrophils in the footpad, the isolated cells were stained with anti-CD45 APC, anti-CD11b FITC, and anti-Ly6G PE antibodies after FcR blocking with an anti-CD16/CD32 antibody. The stained cells were then analyzed using CytoFLEX flow cytometer (Beckman Coulter) in CytExpert (version 2.3) and FlowJo (version 10.5.3) (BD). To quantify the systemic inflammation induced by CFA inoculation, blood samples were collected from the tail vein of mice, and the serum was prepared by centrifugation at 800×*g* for 10 min. The serum concentrations of IL-6 and C5a were measured using ELISA kits according to the manufacturer's instructions.

### 2.4 BCG infection

BCG infection was induced as previously described [31]. Briefly, mice were intratracheally inoculated with 7.5 × 10^6^ CFU of BCG (Tokyo 172 strain). Lung tissues were collected on day 28 post-infection and subjected to histological analyses.

### 2.5 Autoimmune models

To induce experimental autoimmune encephalomyelitis (EAE), female mice were immunized intracutaneously with 200 μg of MOG_35-55_ peptide emulsified in CFA containing 500 μg of heat-killed Mtb. Mice were intraperitoneally injected with 300 ng of pertussis toxin on day 0 and 2. The clinical score of EAE was graded as follows: 0, no disease; 1, limp tail; 2, hind limb weakness; 3, complete hind limb paralysis; 4, hind and forelimb paralysis; and 5, death. For the induction of collagen-induced arthritis (CIA), male mice immunized intracutaneously with 200 μg of chicken type II collagen emulsified in CFA containing 500 μg of heat-killed Mtb. Three weeks after the first immunization, the mice were administered a booster immunization with the same emulsion. The clinical arthritis scores were graded as follows: 0, no swelling; 1, erythema and mild swelling confined to the tarsal joints; 2, erythema and mild swelling extending from the tarsal joint to the digits; 3, erythema and moderate swelling extending from the metatarsal joints; and 4, erythema and severe swelling encompassing the ankle, foot, and digits or ankylosis of the limb (maximal scores = 4). The final score was the sum of the scores of the four paws (maximal score = 16).

### 2.6 Histology

For the histopathological analysis of the spinal cords from EAE-induced mice, the lungs from BCG-infected mice, or the footpads from CFA-injected or DTH-elicited mice, isolated tissues were fixed in 4 % PBS-buffered paraformaldehyde (PFA) (Wako Biochemicals), embedded in paraffin, and 4-μm tissue slices were stained with hematoxylin and eosin (H&E). To evaluate demyelination in EAE-induced mice, spinal cord slices were stained with Luxel fast blue (LFB). For the histopathological analysis of the joints in CIA-induced mice, ankles and knees fixed in 4 % PFA were decalcified in 10 % EDTA (pH 8.0) for two weeks before embedding in paraffin. The tissue slides were analyzed using an all-in-one fluorescence microscope BZ-X700 and BZ-H4XD software (KEYENCE). Bone destruction in the joints of CIA-induced mice was examined using soft X-ray analysis (25 kV, 5 mA, 30 s, SOFRON; SRO- M50, Sofron Inc.).

### 2.7 Recall Th cell response

Draining lymph node (axial and inguinal lymph nodes) cells isolated from EAE- or CIA-immunized mice were stained with anti-mouse CD4 PE and anti-PE Microbeads, and CD4^+^ cells were purified using a magnetic cell sorting system (MACS, Miltenyi Biotec) with an MS column. The CD4^+^ T cells (1 × 10^6^ cells/ml) were co-cultured with irradiated (3000 rad) spleen cells (5 × 10^6^ cells/ml) from C57BL/6N mice in the presence or absence of MOG_35-55_ peptide (20 μg/ml, for EAE) or collagen type II (100 μg/ml, for CIA) in 96-well round bottom plates. The culture supernatants were collected after three days of culturing, and the IFN-γ and IL-17A levels were measured using ELISA kits according to manufacturer's instructions.

### 2.8 In-vitro Th cell differentiation

Whole spleen cells (5 × 10^6^ cells/ml) from C57BL/6N or *Card9*^*−/−*^ mice were cultured with anti-CD3ε (1 μg/ml) and of heat-killed Mtb H37Ra (10 μg/ml) for three days. The BD GoldiStop was added to the culture 6 h before the end of the culture. The cells were collected, fixed, and permeabilized with BD Cytofix/Cytoperm kits, and then stained with anti-CD4 APC, anti-IFN-γ FITC, and anti-IL-17A PE for flowcytometric analysis.

### 2.9 In-vitro cell stimulation

For the stimulation of spleen cells, whole spleen cells or those depleted CD11c-positive cells (2.5 × 10^6^ cells/ml) from WT (C57BL/6N) or *Card9*^*−/−*^ mice were cultured for 16 h in the presence or absence of LPS (50 ng/ml), CpG-ODN (0.5 μM), or heat-killed Mtb H37Ra (10 μg/ml) in 96-well flat bottom plates. To deplete the CD11c-positive cells, whole spleen cells were stained with anti-mouse CD11c PE and anti-PE Microbeads, and CD11c^+^ cells were depleted using a MACS system with an LS column. The levels of IL-6 and IL-12p40 in the culture supernatants were measured by ELISA. For the stimulation of dendritic cells (DCs), pooled dLN cells isolated from WT or *Card9*^*−/−*^ mice at day 6 after intradermal inoculation with IFA or CFA were stained with anti-CD11c PE, anti-MHC-II APC, and anti-SIRPα APC-Cy7 after FcR-blocking with anti-CD16/CD32 antibody. Then, MHCII^+^CD11c^+^SIRPα^hi^ or MHCII ^+^ CD11c ^+^ SIRPα^low^ DCs were purified by a cell sorter (Sony SH800). The purified DCs were stimulated with PMA (50 ng/ml) plus ionomycin (1 μM) for 12 h in 96-well flat bottom plates. Lastly, the levels of IL-6 in the culture supernatants were measured by ELISA.

### 2.10 Adaptive antibody production

Mice were immunized intracutaneously with 200 μg of OVA emulsified in CFA. Four weeks after immunization, blood samples were collected from the tail vein of the OVA-immunized mice, and serum was prepared by centrifugation at 800×*g* for 10 min. For the ELISA detection of OVA-specific immunoglobulin, MaxiSorp 96-well plates (430341, Nunc) were coated with OVA (100 μg/ml in PBS) overnight at 4 °C, washed with 0.05 % Tween-20/PBS three times, and blocked by incubation with 1 % BSA in PBS for 2 h at 37 °C. After washing, serially diluted sera (with 0.5 % BSA in PBS) were added to the OVA-coated plates and incubated for 2 h at room temperature. After washing, HRP-conjugated goat anti-mouse IgM, IgG, IgG1, or IgG2b were added, incubated for 1 h at room temperature, washed, and then detected with TMB reagent by measuring the absorbance at 450 nm.

### 2.11 Delayed type hypersensitivity

Mice were immunized intracutaneously with 200 μg of OVA emulsified in CFA. Seven days after immunization, the mice were challenged subcutaneously at the right footpad with 200 μg of heat-aggregated OVA (70 °C, 1 h) in 20 μl of PBS. Simultaneously, the same volume of PBS was injected subcutaneously into the left footpad of mice as a control. Twenty-four and 48 h after this challenge, footpad thickness was measured using Vernier calipers (Niigata Seiki, VC-15). The DTH response was evaluated as footpad swelling (μm) calculated as follows: footpad swelling = (right footpad thickness of the right footpad) – (thickness of the left footpad).

### 2.12 Statistical analysis

Statistical analyses were performed using GraphPad Prism 5. Comparisons between two groups were performed using a two-tailed unpaired *t*-test. Multiple groups were compared using two-way ANOVA with Bonferroni's post-hoc test. The incidence of EAE and CIA was analyzed using Kaplan-Meier curves and log-rank tests. *P* < 0.05 was considered statistically significant.

## 3 Results

### 3.1 Card9 deletion attenuates tissue-damaging inflammation by mycobacterial adjuvants

**Fig. 1:**
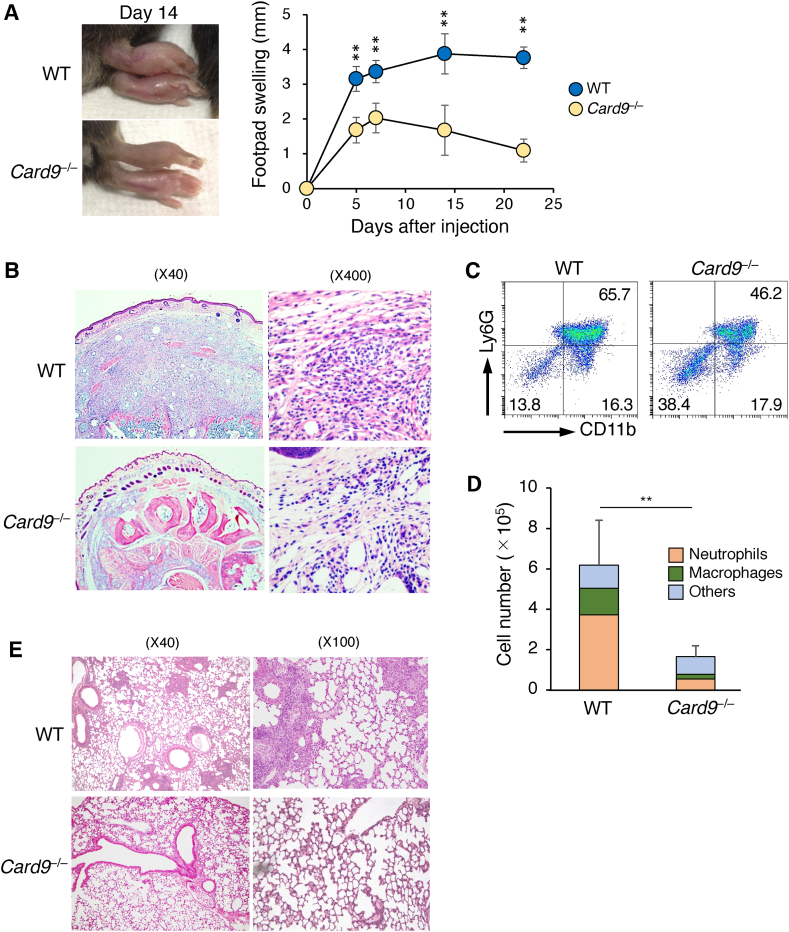
Card9 deletion attenuates the tissue-damaging inflammation induced by mycobacterial adjuvants. (A) Time course of footpad swelling in WT control and *Card9*^*−/−*^ mice (n = 8) after subcutaneous injection of CFA. Footpad swelling was indicated by increased thickness in the pre-inoculation controls of each mouse. Photographs of the footpads on day 14 are shown. (B) Representative hematoxylin and eosin (H&E)-stained sections from day 14. (C) Flow cytometry of infiltrated neutrophils (CD45^+^CD11b^+^Ly6G^+^), macrophages (CD45^+^CD11b^+^Ly6G^–^), and other leukocytes (CD45^+^CD11b^–^) from the footpads on day 14. (D) Average cell numbers (n = 4) were calculated from the data in C. (E) Representative H&E-stained sections of the lung lobes on day 28 from WT and *Card9*^*−/−*^ mice intratracheally infected with 5 × 10^6^ CFU of BCG. Data in A and D are presented as mean ± SEM and are representative of three independent experiments. Statistical significance was analyzed using a two-way ANOVA followed by Bonferroni's test (A) or a two-tailed unpaired *t*-test (D). ∗∗*p* < 0.01 vs. WT.

To evaluate the impact of Card9 deletion on the inflammatory response evoked by mycobacterial adjuvants at the site of inoculation, we subcutaneously injected CFA containing heat-killed Mtb into the footpads of *Card9*^*−/−*^ and WT control mice. In the WT mice, CFA inoculation caused a progressive increase in edema of the footpads, peaking at day 14 after inoculation and lasting for more than three weeks without regression (Fig. 1A). However, *Card9*^*−/−*^ mice exhibited significantly reduced edema of the footpads, which was relieved much earlier than that in WT mice. Histopathological analysis of the footpads on day 14 revealed severe cellulitis-like inflammation manifested as neutrophil infiltration throughout the tissue in the WT mice (Fig. 1B). By contrast, inflammation was much milder and cellulitis was virtually absent in *Card9*^*−/−*^ mice (Fig. 1B). Flow cytometric analyses of the infiltrating cells revealed that CD11b^+^Ly6G^+^ neutrophils were predominant and CD11b^+^Ly6G^–^ macrophages were subdominant in WT mice, whereas the number of these cells markedly decreased in *Card9*^*−/−*^ mice (Fig. 1C and D). These results suggest that Card9 deletion significantly ameliorated CFA-induced tissue-damaging local inflammation.

Live attenuated BCG is widely used as a vaccine against tuberculosis and as an adjunct therapy for superficial bladder cancer. This bacterium is rapidly eliminated by the cellular immune system in immunocompetent hosts. However, it forms granulomatous lesions characterized by macrophage accumulation when inoculated into the lungs of animals, even at the time point when the bacilli are eliminated from the tissue. We examined the effect of Card9 deletion on lung granuloma formation induced by intratracheal inoculation with a vaccine strain of BCG (Tokyo 172). While the WT control mice exhibited massive granulomatous lesions in the infected lungs on day 28 after inoculation, similar lesions were virtually absent in the lungs of *Card9*^*−/−*^ mice, with only a few dispersed inflammatory cell accumulations (Fig. 1E). These results suggest that Card9 signaling is vital for eliciting local tissue-damaging inflammation induced by mycobacterium-containing vaccines.

### 3.2 Card9 deletion prevents autoimmunity inducted by immunization with mycobacterial adjuvants

**Fig. 2:**
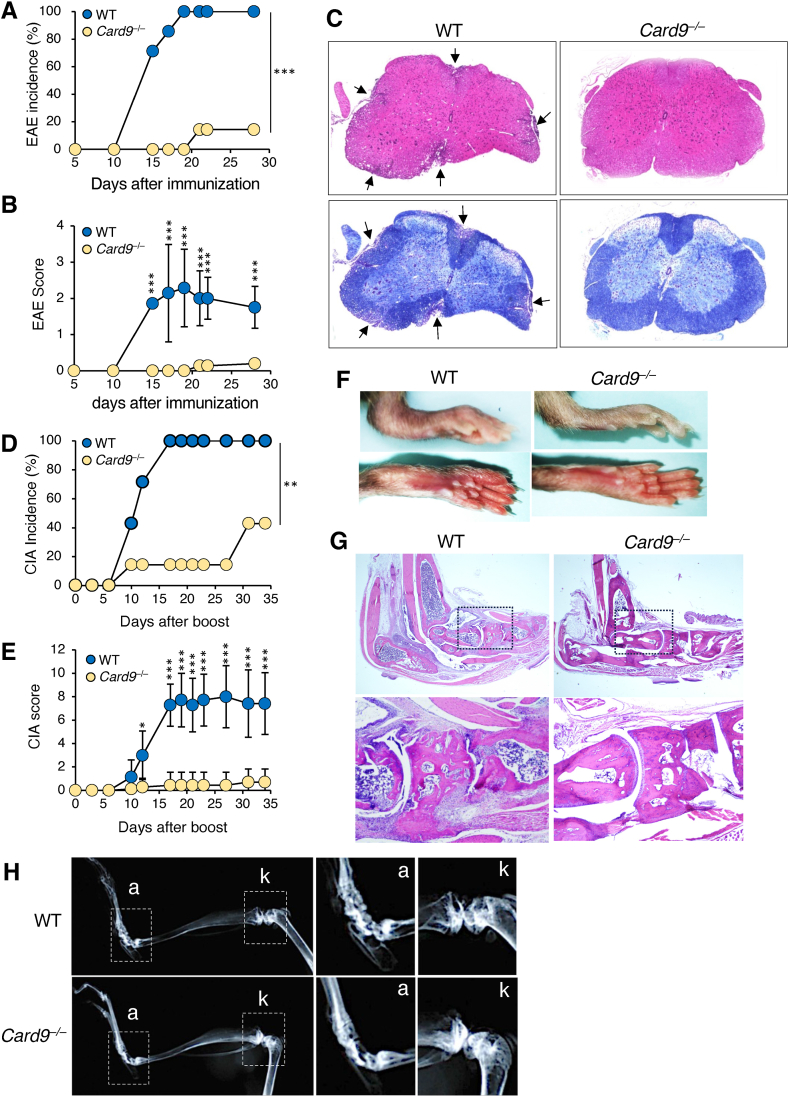
Card9 deletion prevents autoimmunity induced by immunization with mycobacterial adjuvants. (A–C) Experimental autoimmune encephalomyelitis (EAE) was induced in WT and *Card9*^*−/−*^ mice (n = 9) by the inoculation of MOG_35-55_ peptide with CFA. (A and B) Kaplan–Meier plot of the incidence (A) and time course of clinical scores (B) of EAE. (C) Representative H&E (Upper) and Luxol fast blue (lower) staining of spinal cord sections at day 21. (D–H) Collagen-induced arthritis (CIA) was induced in WT and *Card9*^*−/−*^ mice (n = 7) by the inoculation of collagen type II with CFA. (D and E) Kaplan–Meier plot of the incidence (D) and time course of clinical scores (E) of CIA. (F–H) Representative pictures of swelling of the ankles and toes (F) and H&E-stained sections of the ankles (G), and X-ray radiographs (H) of the ankles (a) and knees (k) on day 34 after boost immunization. Data in B and E are presented as mean ± SEM and are representative of two independent experiments. Statistical significance was analyzed by Kaplan-Meier estimates and log-rank test (A and D) or by two-way ANOVA followed by Bonferroni's test (B and E). ∗*p* < 0.05, ∗∗*p* < 0.01, ∗∗∗*p* < 0.005. (For interpretation of the references to colour in this figure legend, the reader is referred to the Web version of this article.)

Autoimmunity is a characteristic of tuberculosis [17]. In addition, immunizing animals with autoantigens using Mycobacterium adjuvants can destroy their self-tolerance and lead them to develop autoimmunity to the immunized antigens. To evaluate the impact of Card9 deficiency on autoimmune induction by mycobacterial adjuvants, we established two mouse autoimmune models, myelin oligodendrocyte glycoprotein (MOG)-induced experimental autoimmune encephalomyelitis (EAE) and collagen II-induced arthritis (CIA), induced by immunization with CFA. In the EAE model, the clinical signs of EAE (i.e., paralysis of the hindlimbs or abnormal locomotion) appeared in the WT mice from approximately two weeks after immunization, with 100 % of the mice eventually developing EAE by day 19 (Fig. 2A). However, only one out of nine *Card9*^*−/−*^ mice showed signs of EAE from day 21, with a significantly lower clinical score (1) than the mean score of WT mice (2.3 ± 0.8) (Fig. 2B). Histopathological analysis using hematoxylin and eosin (H&E) and Luxol Fast Blue (LFB) staining showed that all WT mice had significant inflammatory cell infiltration and concomitant demyelination in the spinal cord (Fig. 2C). In contrast, no obvious histopathological abnormalities were observed in the *Card9*^*−/−*^ mice. In the CIA model, clinical signs of arthritis (i.e., redness and swelling in the hind paws and finger joints) appeared seven days after booster immunization, leading to a 100 % incidence by day 16 in the WT mice (Fig. 2D and F). In contrast, the *Card9*^*−/−*^ mice developed arthritis with significantly lower cumulative incidence and delayed onset than the WT mice, and the mean clinical score was significantly lower than that of the WT mice (Fig. 2D and E). In correlation with the clinical findings, histopathological and X-ray analyses showed that synovial inflammation, pannus formation (Fig. 2G), and cartilage and bone destruction (Fig. 2H) observed in the WT mice were not observed in the joints of the *Card9*^*−/−*^ mice. These results suggested that Card9-mediated innate immune activation is mandatory for autoimmune induction by mycobacterial adjuvants.

### 3.3 Card9 is required for the early development of pathogenic Th17

**Fig. 3:**
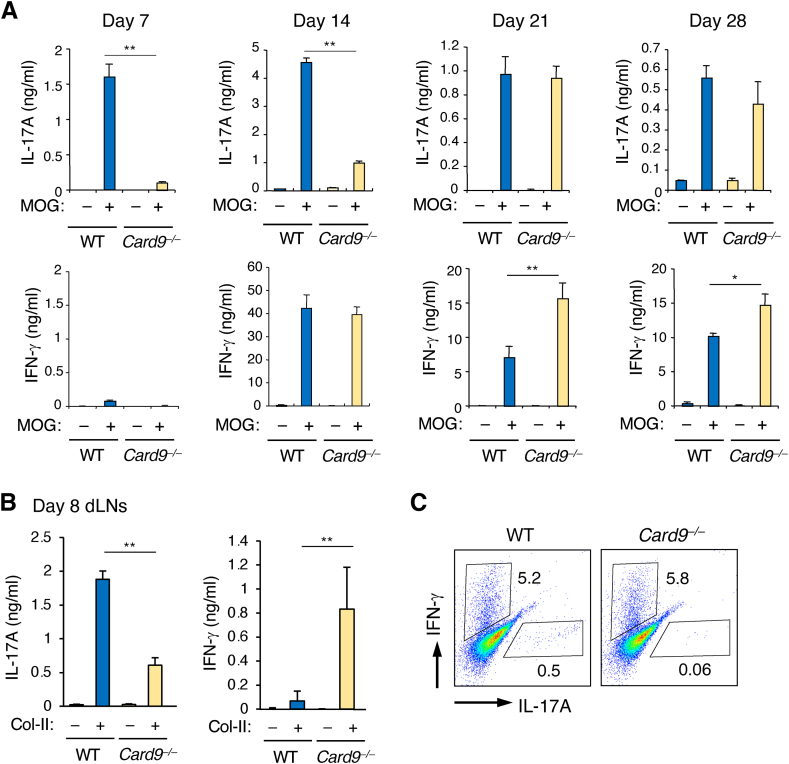
Card9 is required for the early development of pathogenic Th17 during the induction of autoimmunity. (A) Development of pathogenic Th1 and Th17 cells during EAE induction. CD4^+^ T cells isolated from the draining lymph nodes of WT and *Card9*^*−/−*^ mice on day 7, 14, 21 and 28 after EAE immunization were stimulated *ex vivo* with MOG_35-55_ peptide for three days. The levels of IL-17A and IFN-γ in the culture supernatants were measured by ELISA. (B) Development of pathogenic Th1 and Th17 cells during CIA induction. CD4^+^ T cells isolated from the draining lymph nodes of WT and *Card9*^*−/−*^ mice on day 8 after boosting immunization of CIA were stimulated *ex vivo* with collagen type II (Col-II) for three days. The levels of IL-17A and IFNγ in the culture supernatants were measured by ELISA. (C) Flow cytometry of *in vitro*-developed Th1 and Th17 cells. Whole spleen cells from WT and *Card9*^*−/−*^ mice were cultured for three days in the presence of anti-CD3ε plus heat-killed Mtb H37Ra. The cells were fixed and analyzed by flowcytometry for intracellular expression of IFN-γ and IL-17A to assess the development of Th1 and Th17 cells, respectively. Data in A and B are presented as mean ± SEM and are representative of three independent experiments. Statistical significance was analyzed by two-tailed unpaired *t*-test (A and B). ∗*p* < 0.05, ∗∗*p* < 0.01.

Since Th1 and Th17 cells play pivotal roles in the pathogenesis of EAE and CIA [18], we investigated the recall cytokine response of CD4^+^ T cells from draining lymph nodes (dLNs) of EAE-immunized mice to the MOG_35-55_ peptide *ex vivo*. Unexpectedly, IL-17A production was comparable between the WT and *Card9*^*−/−*^ CD4^+^ T cells on day 21 and 28 after immunization (Fig. 3A). Moreover, IFNγ production of the same cells was even significantly increased by the *Card9* deficiency. We then investigated Th cells at earlier time points (day 7 and 14) and found that IL-17A production was remarkably reduced in the *Card9*^*−/−*^ CD4^+^ T cells compared to the WT cells. In contrast, IFN-γ production was comparable between the WT and *Card9*^*−/−*^ CD4^+^ T cells from the day 14 dLNs and undetectable in both cells from the day 7 dLNs. Similarly, a lower production of IL-17A was observed in the *Card9*^*−/−*^ CD4^+^ T cells from the day 8 dLNs of CIA-immunized mice (Fig. 3B). To further verify the impact of Card9 on Th cell development, we stimulated whole spleen cells from WT and *Card9*^*−/−*^ mice with anti-CD3ε mAb in the presence of heat-killed Mtb for four days, followed by the staining of intracellular IFNγ and IL-17A in CD4^+^ T cells to detect Th1 and Th17 cells, respectively, by flow cytometry. While the ratio of IFNγ-producing CD4^+^ T cells was comparable between the cultures of WT and *Card9*^*−/−*^ cells, that of IL-17A-producing CD4^+^ T cells was remarkably reduced in the culture of the *Card9*^*−/−*^ cells (Fig. 3C). These results suggest that the resistance of *Card9*^*−/−*^ mice to mycobacterium-induced autoimmunity is likely due to the defective development of pathogenic Th17, but not Th1, in the early phase of immunization.

### 3.4 Card9 is required for the development of IL-6-producing Sirpα^high^ dendritic cells

**Fig. 4:**
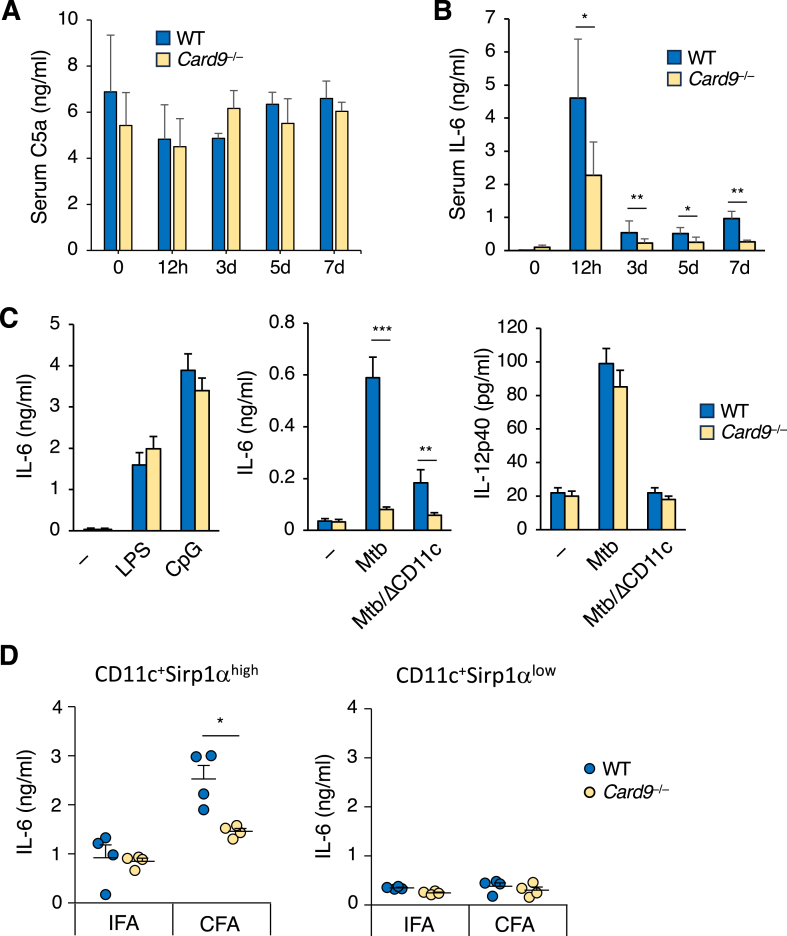
Card9 is required for the development of IL-6-producing Sirpα^high^ dendritic cells. Complement C5a (A) and IL-6 (B) levels in the sera of WT and *Card9*^*−/−*^ mice after dermal CFA inoculation. (C) IL-6 or IL-12p40 production by WT and *Card9*^*−/−*^ splenocytes or those depleted for CD11c^+^ cells (ΔCD11c) stimulated with LPS, CpG, or heat-killed Mtb for 16 h. (D) Analysis of IL-6-producing Sirpα^high^ dendritic cells in the dLNs. CD11c ^+^ MHC-II^high^Sirpα^high^ or CD11c ^+^ MHC-II^high^Sirpα^low^ dendritic cells were isolated by FCM-sorting from dLNs of WT and *Card9*^*−/−*^ mice at day 6 after IFA or CFA inoculation and stimulated with PMA plus ionomycin for 12 h. The culture supernatants were analyzed for the level of IL-6 by ELISA. Data are presented as mean ± SEM and are representative of at least three independent experiments. Statistical significance was analyzed by two-way ANOVA followed by Bonferroni's test (A and B) or two-tailed unpaired *t*-test (C and D). ∗*p* < 0.05, ∗∗*p* < 0.01, ∗∗∗*p* < 0.005.

The complement system has been suggested to be involved in the pathogenesis of autoimmune diseases [19,20]. On this basis, we investigated whether the deletion of *Card9* affected complement activation. However, elevation of C5a, an indicator of complement activation, was not clearly observed in the sera of neither the WT nor *Card9*^*−/−*^ mice for at least seven days following dermal CFA inoculation (Fig. 4A). This suggests that complement activation is unlikely to be responsible for the reduced induction of autoimmunity associated with defective early Th17 development in *Card9*^*−/−*^ mice. Because IL-6 is a dominant factor that primes pathogenic Th17 cell differentiation [21], we examined the impact of Card9 deficiency on IL-6 induction by mycobacterial adjuvants. Subcutaneous inoculation with CFA increased the serum IL-6 levels, which peaked 12-h after inoculation and declined thereafter, but persisted for several days in WT mice (Fig. 4B). The *Card9*^*−/−*^ mice showed significantly lower serum IL-6 levels than the WT mice throughout the observation period, indicating the ability of *Card9*^*−/−*^ immune cells to produce IL-6 in response to mycobacterial PAMPs. We stimulated WT and *Card9*^*−/−*^ splenocytes *ex vivo* with LPS, CpG-ODN, or heat-killed Mtb and examined the production of IL-6 and IL-12p40, common components of IL-12 and IL-23, which are essential cytokines for Th1 and Th17 differentiation, respectively. The *Card9*^*−/−*^ splenocytes produced comparable levels of IL-6 in response to LPS or CpG-ODN, but produced remarkably lower levels of IL-6 in response to Mtb (Fig. 4C, left). In contrast, the loss of Card9 did not affect IL-12p40 production in response to Mtb infection (Fig. 4C, middle and right panels). The depletion of CD11c^+^ cells from splenocytes almost completely abolished both IL-6 and IL-12p40 production, indicating that dendritic cells (DCs) were the main cellular source of these cytokines. Heink et al. found that a subset of DCs positive for the signaling regulator Sirpα was essential for the generation of pathogenic Th17 cells in EAE induction by trans-presenting IL-6 to T cells during the process of cognate T-DC interaction in the dLNs [22]. Thus, we investigated the impact of Card9 deficiency on the development of IL-6-producing Sirpα^+^ DCs after immunization of CFA. Consistent with Heink et al.’s findings, Sirpα^high^ DCs bearing the capacity to produce IL-6 were increased in the dLNs of WT mice after immunization with CFA, but not with incomplete Freund's adjuvant (IFA) lacking heat-killed Mtb (Fig. 4D, left), whereas Sirpα^low^ DCs had no capacity to produce IL-6 (Fig. 4D, right). However, the *Card9*^*−/−*^ mice showed significantly reduced development of IL-6-producible Sirpα^high^ DCs in the dLNs. Together, these results suggest that Card9 is required for the induction of IL-6-producing Sirpα^high^ DCs, which are essential to prime pathogenic Th17, in the dLN after immunization with mycobacterial adjuvants.

### 3.5 Card9 is dispensable for the induction of adaptive humoral and type-I cellular immunity

**Fig. 5:**
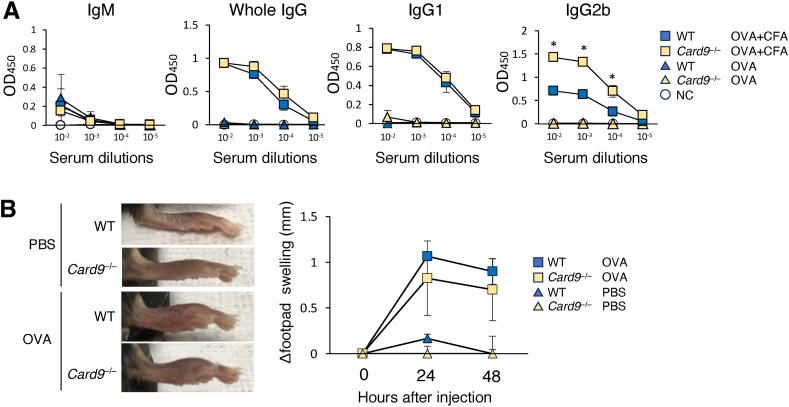
Card9 is dispensable for the induction of adaptive humoral and type-I cellular immunity. (A) Normal adaptive humoral response in *Card9*^*−/−*^ mice. WT and *Card9*^*−/−*^ mice (n = 8) were intradermally injected with OVA alone or CFA-emulsified OVA. Four weeks after the immunization, the serum titers of OVA-specific IgM, whole IgG, IgG1 and IgG2b antibodies were determined by ELISA. NC indicates data from nonimmunized WT control mice. (B) Normal DTH reaction in *Card9*^*−/−*^ mice. WT and *Card9*^*−/−*^ mice (n = 8) immunized with CFA-emulsified OVA were challenged with OVA into the right footpads or with control PBS into the left footpads, and footpad swelling was measured at 24 h and 48 h after the challenge. Photographs of the footpads at 24 h after OVA or PBS challenge are shown. Footpad swelling was calculated as follows: footpad swelling = (right footpad thickness of the right footpad) – (thickness of the left footpad). Data are presented as mean ± SEM and are representative of at least three independent experiments. Statistical significance was analyzed by two-way ANOVA followed by Bonferroni's test. ∗*p* < 0.05.

The above results indicate that the detrimental effects of mycobacterial adjuvants can be eliminated by Card9 deletion. Next, we investigated whether the loss of Card9 affects the development of adaptive humoral immunity and Th1-mediated cellular immunity after immunization with mycobacterial adjuvants. The immunization of WT and *Card9*^*−/−*^ mice with ovalbumin (OVA) in CFA resulted in elevated levels of OVA-specific whole IgG antibodies in the serum compared with immunization with OVA alone (Fig. 5A). An analysis of IgG1 (Th2-mediated) and IgG2b (Th1-mediated) subtypes revealed that the loss of Card9 did not affect IgG1 titers, but increased IgG2b titers in the sera compared to WT mice, indicating an enhanced Th1-mediated humoral response in *Card9*^*−/−*^ mice. To investigate antigen-specific cellular immune responses, we performed a delayed type-hypersensitivity (DTH) reaction induced by a Th1-mediated type I immune response [23]. After immunization with OVA emulsified with CFA, the WT and *Card9*^*−/−*^ mice were challenged with an OVA injection into the footpads, and swelling was measured for 48 h. As a result, a comparable DTH reaction was observed between the WT and *Card9*^*−/−*^ mice (Fig. 5B). These results suggest that both adaptive humoral and type I cellular responses induced by Mycobacterium adjuvants are unaffected in the absence of Card9.

## 4 Discussion

Components derived from Mtb and acid-fast bacteria have been reported to show remarkable immunogenicity, stimulating potent adaptive immune responses in vaccination and immunotherapy settings [[3], [4], [5], [6], [7]]. This is because of their ability to activate innate immune cells, such as macrophages and dendritic cells, leading to robust local inflammation and subsequent adaptive immune responses through antigen presentation. These adjuvants can induce a durable immune memory, providing prolonged protection against targeted pathogens or cancer cells. This feature is particularly advantageous for the development of vaccines and immunotherapies requiring sustained immune activation. Although these adjuvants have proven effective, they have also raised safety concerns owing to their potential to induce more pronounced local and systemic side effects than other adjuvants [9]. This reactogenicity may limit their use in certain patient populations or may require careful dose optimization. One approach to address these limitations is to develop modified or synthetic adjuvants that retain their immunostimulatory properties while minimizing adverse effects. By tailoring these adjuvants, one could unlock their full potential in clinical applications.

In this study, we found that the loss of Card9 significantly alleviated local and systemic inflammation at the injection site and reduced the incidence of CFA-induced autoimmunity. Loss of Card9 impaired the early development of pathogenic Th17 but not Th1 cells during autoimmune induction, likely due to the defective development of IL-6-producing CD11c ^+^ Sirpα^high^ DCs in the dLNs, which are required for the priming of pathogenic Th17. However, loss of Card9 did not affect the induction of antigen-specific humoral or Th1-mediated cellular immunity.

Mycobacteria contain various PAMPs that stimulate and modulate host innate immune receptors [24]. For example, lipoproteins/lipoglycans and muramyl dipeptide (MDP) in the peptidoglycan layer are recognized by TLR2 and NLR NOD2, respectively. TLR9 and cGAS sense the endosomal and cytosolic bacterial DNAs, respectively. The CLRs mannose receptor (MR), DC-SIGN, and Dectin-2 recognize the glycolipid Mannose-capped LAM (Man-LAM) in the cell walls. The CLRs Mincle and MCL recognize TDM and mediate TDM-induced toxic immune responses. Card9 has been shown to play an essential role in activating myeloid cells through the FcRγ-chain associated CLRs such as Dectin-2, Mincle, and MCL [13]. It is important to determine which of these components and host receptors are responsible for beneficial or undesirable responses to mycobacterial adjuvants via Card9. Shenderov et al. investigated innate immune receptors activated by CFA to promote Th17 polarization and found that IL-1β/IL-1R signaling played a pivotal role in driving it [25]. Biochemical fractionation studies revealed that peptidoglycan fraction from heat-killed mycobacteria in CFA mainly contributes to inflammasome activation for the processing of pro-IL-1β, while Mincle and Card9 play a major role in triggering pro-IL-1β expression. Immunization with purified peptidoglycan and TDM in mineral oil synergistically recapitulated the Th17-promoting activity of CFA [25]. The importance of Card9 and Mincle in the development of autoimmunity has been proven in animal models of uveitis and experimental autoimmune uveoretinitis (EAU) induced by immunization with CFA [26]. This report also showed that Card9 and Syk, an essential tyrosine kinase for signal activation downstream of ITAM-coupled CLRs, were responsible for Th17 polarization and Th17-associated, but not Th1-associated, antigen-specific responses, which is consistent with the findings on EAE reported in the current study. These findings suggest that the Card9-induced Th17 development is likely initiated through FcRγ-associated CLRs represented by Mincle. Therefore, blocking these receptors or their ligands from mycobacterial adjuvants may recapitulate the absence of Card9 and remove the harmful facets of the adjuvants.

Macrophages and dendritic cells (DCs) play key roles in both innate and adaptive immune responses by producing cytokines. The differentiation of Th17 cells is determined by TGF-β and IL-6 and promoted by IL-1. In the current study, we found that Card9 signalling was also required for the development of IL-6-producing Sirpα^+^ DCs, which are known to be essential for the induction of pathogenic Th17 cells in autoimmunity [22]. Th17 and IL-17 have been linked to resistance to infection by fungi, such as *Candida albicans* [27]. Hypomorphic Card9 mutations have also been identified in familial mycosis patients [14]. In these patients, the number of Th17 cells in the blood significantly decreased. Gross et al. found that *C. albicans* engaged Dectin-1 in DCs to secrete IL-1β in a Syk and Card9-dependent manner, leading to the development of protective Th17 immunity against the fungus [15,28]. Thus, Card9 may also promote pathogenic Th17 development through IL-1β secretion by DCs in the vaccination with CFA.

Mycolic acids (MAs) are lipids unique to the genus Mycobacteria and its related species. They are expressed on the surface of mycobacteria in glycosylated or non-glycosylated forms and their abundance and proportion affect the host immune response [29]. We previously reported that glycosylated forms of MAs, such as TDM and glucose-monomycolate (GMM), induce nitric oxide (NO)-producing M1-like macrophages in a Mincle- and Card9-dependent manner [30]. In contrast, the non-glycosylated forms of MAs, such as free mycolate and glycerol-mono-mycolate, were recognized by triggering the receptor expressed on myeloid cells 2 (TREM2) and inducing permissive macrophages to produce the macrophage chemoattractant MCP-1 in a Card9-independent manner, but did not produce NO and TNF. Interestingly, loss of TREM2 resulted in exacerbated TDM-induced inflammation, such as lung granuloma formation and thymic atrophy, through the hyperactivation of Mincle, indicating a suppressive role for TREM2 and its MA ligands in the innate immune response to mycobacteria. However, Kubota et al. reported that vaccination with purified free MAs as adjuvants could induce substantial antigen-specific humoral and cellular immune responses and prevent the growth of antigen-expressing tumors as effectively as vaccination with CFA [31]. Similar to CFA vaccination in the absence of Card9, the Th1 immune response, rather than Th17, was induced by MA. Despite its immunogenicity, tissue-destructive inflammation with neutrophil infiltration at the injection site induced by CFA was not observed in MA-injected mice. Therefore, MA could be used as a seed adjuvant for the development of novel vaccines with fewer adverse effects. We observed that both the adaptive humoral and type I cellular responses to antigens immunized with CFA were unaffected in the absence of Card9. Therefore, it is likely that Card9 is dispensable for the efficacy of vaccines, such as antiviral and anticancer vaccines, in which the type I immune response plays a leading role in target eradication. Indeed, we previously reported that the loss of Card9 did not compromise antiviral humoral and cellular immunity but significantly attenuated pneumonia and reduced mortality in influenza-infected mice [32]. Although outside the scope of the present study, there is also a need to test whether Card9 deficiency affects the efficacy of vaccines containing mycobacterial adjuvants in preventing intracellular pathogens and cancers.

In conclusion, our findings suggest that through the addition of Card9 signal inhibitors or the depletion of ligands for Card9-activating receptors from mycobacterial components, we may be able to develop safer adjuvants suitable for vaccines against intracellular pathogens and cancers that are primarily protected by Th1-mediated immunity.

## Ethics declarations

The study protocol was reviewed and approved by the Animal Research Committee of Kagoshima University, with approval No. MD16114.

## Data availability statement

Data will be made available on request.

## Funding

This work was supported by JSPS KAKENHI (grant number 22021034 and 20060019 (H.H.), 21K08160 (H.I.), 2686092 (E.I.), and 23K07606 (H.M.)), 10.13039/100007428The Naito Foundation (H.H.), 10.13039/100007449Takeda Science Foundation (H.H.), The 10.13039/100008732Uehara Memorial foundation (H.H.), Japan Rheumatism Foundation (H.H.), and Practical Research Project for Allergic Diseases and Immunity 20ek0410052 (H.I.) from the 10.13039/100009619Japan Agency for Medical Research and Development.

## CRediT authorship contribution statement

**Hideo Mitsuyama:** Writing – original draft, Investigation. **Ei'ichi Iizasa:** Writing – review & editing, Methodology, Investigation. **Akiko Kukita:** Investigation. **Shuji Toda:** Investigation. **Hiroki Yoshida:** Project administration. **Hiromasa Inoue:** Project administration. **Hiromitsu Hara:** Writing – review & editing, Writing – original draft, Supervision, Conceptualization.

## Declaration of competing interest

The authors declare that they have no known competing financial interests or personal relationships that could have appeared to influence the work reported in this paper.
